# IbINV Positively Regulates Resistance to Black Rot Disease Caused by *Ceratocystis fimbriata* in Sweet Potato

**DOI:** 10.3390/ijms242216454

**Published:** 2023-11-17

**Authors:** Dongjing Yang, Xiaofeng Bian, Ho Soo Kim, Rong Jin, Fangyuan Gao, Jingwei Chen, Jukui Ma, Wei Tang, Chengling Zhang, Houjun Sun, Yiping Xie, Zongyun Li, Sang-Soo Kwak, Daifu Ma

**Affiliations:** 1 Key Laboratory of Biology and Genetic Improvement of Sweetpotato, Ministry of Agriculture and Rural Affairs, Xuzhou Institute of Agricultural Sciences in Jiangsu Xuhuai District, Xuzhou 221131, China; njnd831215@126.com (D.Y.); jinrong_2012@126.com (R.J.); fygao199503@163.com (F.G.); ibcjw0825@126.com (J.C.); majukui@126.com (J.M.); tangv0001@163.com (W.T.); zhchling5291@163.com (C.Z.); sunhouj1980@163.com (H.S.); xieyiping6216@163.com (Y.X.); 2 Institute of Food Crops, Jiangsu Academy of Agricultural Sciences, Nanjing 210014, China; bianxiaofeng2@163.com; 3 Plant Systems Engineering Research Center, Korea Research Institute of Bioscience and Biotechnology (KRIBB), 125 Gwahak-ro, Daejeon 34141, Republic of Korea; hskim@kribb.re.kr; 4 College of Life Science, Jiangsu Normal University, Xuzhou 221116, China; zongyunli@jsnu.edu.cn

**Keywords:** sweet potato, invertase, plant growth, black rot disease, sugar metabolism

## Abstract

Black rot disease, caused by *Ceratocystis fimbriata* Ellis & Halsted, severely affects both plant growth and post-harvest storage of sweet potatoes. Invertase (INV) enzymes play essential roles in hydrolyzing sucrose into glucose and fructose and participate in the regulation of plant defense responses. However, little is known about the functions of INV in the growth and responses to black rot disease in sweet potato. In this study, we identified and characterized an *INV-like* gene, named *IbINV*, from sweet potato. IbINV contained a pectin methylesterase-conserved domain. *IbINV* transcripts were most abundant in the stem and were significantly induced in response to *C. fimbriata*, salicylic acid, and jasmonic acid treatments. Overexpressing *IbINV* in sweet potato (OEV plants) led to vigorous growth and high resistance to black rot disease, while the down-regulation of *IbINV* by RNA interference (RiV plants) resulted in reduced plant growth and high sensitivity to black rot disease. Furthermore, OEV plants contained a decreased sucrose content and increased hexoses content, which might be responsible for the increased INV activities; not surprisingly, RiV plants showed the opposite effects. Taken together, these results indicate that IbINV positively regulates plant growth and black rot disease resistance in sweet potato, mainly by modulating sugar metabolism.

## 1. Introduction

Sweet potato (*Ipomoea batatas* (L.) Lam.) is an important food crop cultivated worldwide, particularly in developing countries [1,2]. China is the largest sweet potato producer in the world, and sweet potato occupies a very important position in China’s national economy. Because of its high yield potential and wide range of adaptability to marginal lands, sweet potato can meet the food and nutrition security needs for sustainable development in the 21st century [3]. According to the latest statistics of the Food and Agriculture Organization (FAO), the current sweet potato harvested area is about 2.2 × 10^6^ hm^2^, which accounts for about 30% of the world’s total arable land area. The emerging research shows that eating sweet potato can reduce one’s level of blood sugar and blood pressure and prevent overweight [4,5]. Sweet potato tubers are rich in nutrients such as dry matter, beta-carotene, vitamins (C, B, and E), starch and protein, and minerals such as potassium, phosphorus, zinc, copper, iron, manganese, and calcium, which are important for good health [6,7]. Sweet potato is not only a food for humans and animals, but it is also an important crop that provides sufficient raw materials for the development of starch, fermentation, natural pigments, and other processing industries [8].

During the field and storage periods, many fungi and bacterial diseases could cause severe damage to sweet potato. Black rot of sweet potato, caused by *Ceratocystis fimbriata* Ellis & Halsted, is one of the main sweet potato fungal diseases, and was first identified in 1890 [9]. This pathogen can infect not only the stem but also the storage root, causing the death of seedlings and rotting in storage cellar. *C. fimbriata* also has a wide range of hosts, including sweet potato, coffee, rubber tree, yerba mate, and so on [10,11,12]. Previous reports showed that black rot occurred in all sweet potato-producing areas in the world [13]. In China, black rot is common in all sweet potato-producing areas. The yield loss caused by sweet potato black rot is about 5–10% every year, and it can reach 20–50% or even higher when the damage is serious [14]. In addition, it can produce furanoterpenoids toxins in the storage roots after being infected by black rot disease, which leads to poisoning or even death if eaten by people or animals [15]. Black rot disease can spread far away by the transportation of seedlings or storage roots, so it has been listed as a domestic quarantine object in China.

Although the loss caused by *C. fimbriata* can be severe, there has been very limited research about the controlling strategies of this pathogen. A previous study showed that the electronic nose could be used for early discrimination and prediction of *C. fimbriata*-infected sweet potato before symptoms were observed [14]. In order to control black rot disease in sweet potato, some researchers tried to develop different biological control methods. It was reported that chitosan could effectively control the *C. fimbriata* development in sweet potato storage root [16]. Fumigation with *Pseudomonas chlororaphis* subsp. SPS-41 could exert antifungal activity by inducing oxidative stress and mitochondrial dysfunction in *C. fimbriata* [17]. It was indicated that antifungal volatile organic compounds from different kinds of microbial strains could control the black rot disease of sweet potato [18,19,20,21,22]. However, these biological agents have not been industrialized; therefore, they could not be widely applied. Some chemicals have also been used to control black rot disease. Mohsin et al. investigated the efficacy of tebuconazole (TEB) and trifloxystrobin (TRI) against *C. fimbriata*, and the result showed that TEB and TRI could control black rot disease in sweet potato by inhibiting the advancement of *C. fimbriata* [23]. Preventive applications of quinolinic acid could significantly reduce the disease incidence of *C. fimbriata* on sweet potato, achieving higher control efficacy in comparison with the commercial fungicides prochloraz and carbendazim [24]. Despite these advances, there are currently only five chemicals registered in China for controlling the black rot disease of sweet potato, including amobam, allicin, ethylicin, thiophanate-methyl, and carbendazim. The use of some of the fungicides mentioned is restricted due to their negative effects on human health and the environment [25]. Elevating host resistance could be a desirable way to relieve crop loss caused by plant pathogens. The development of sweet potato varieties, which can be both resistant to black rot disease and produce high yields, has not been as challenging as traditional breeding. However, it takes a very long time, and knowledge of the genetic basis of disease resistance in sweet potato is limited [26].

More and more studies have demonstrated that genetic manipulation is a potential approach to improving the disease resistance of sweet potato. In this field, overexpressed *IbBBX24*, thionin, and *IbSWEET10* enhanced the disease resistance of sweet potato plants [27,28,29]. As we know, plant invertases (INVs) are sucrolytic enzymes that irreversibly catalyze the hydrolysis of sucrose to glucose and fructose and are essential for the regulation of carbohydrate metabolism, plant growth, and plant defense responses [30]. INVs can be grouped into two subfamilies: acidic invertases, including the cell wall invertases (CWINs) and the vacuolar invertases (VINs), and the neutral/alkaline invertases, which are localized in the cytoplasm, mitochondria, or plastid according to pH optima and solubility characteristics [31]. CWINs and VINs have similar enzymology and biochemical characteristics, as well as high homology. The first invertase gene in higher plants was successfully cloned from carrots in 1990 [32]. Various invertase genes have been cloned from many plants, and their biochemical characteristics, physiological functions, and biological regulation have been deeply studied. Plant INVs play important roles in reproductive and vegetative development, and their activity is usually regulated at the post-translational level [33]. For instance, plant invertase inhibitors (INHs) can directly target the INV’s active site and inhibit the activity of INVs [34,35]. However, little is known about the regulation of INV-mediated sucrose metabolism and disease resistance responses in sweet potato.

In this study, we identified an *INV*-like gene (named *IbINV*) from sweet potato (cv. Xushu29). An expression pattern analysis of *IbINV* in sweet potato was performed. Furthermore, its roles in black rot disease resistance in transgenic sweet potato plants, both by overexpression and RNA interference, were investigated. Our results indicated that IbINV positively regulated plant growth and black rot disease resistance. This study will lay the foundation for further understanding of the function of *IbINV* and its application in improving the disease resistance of sweet potato through genetic manipulation.

## 2. Results

### 2.1. Cloning and Characterization of Sweet Potato IbINV Gene

**Figure 1:**
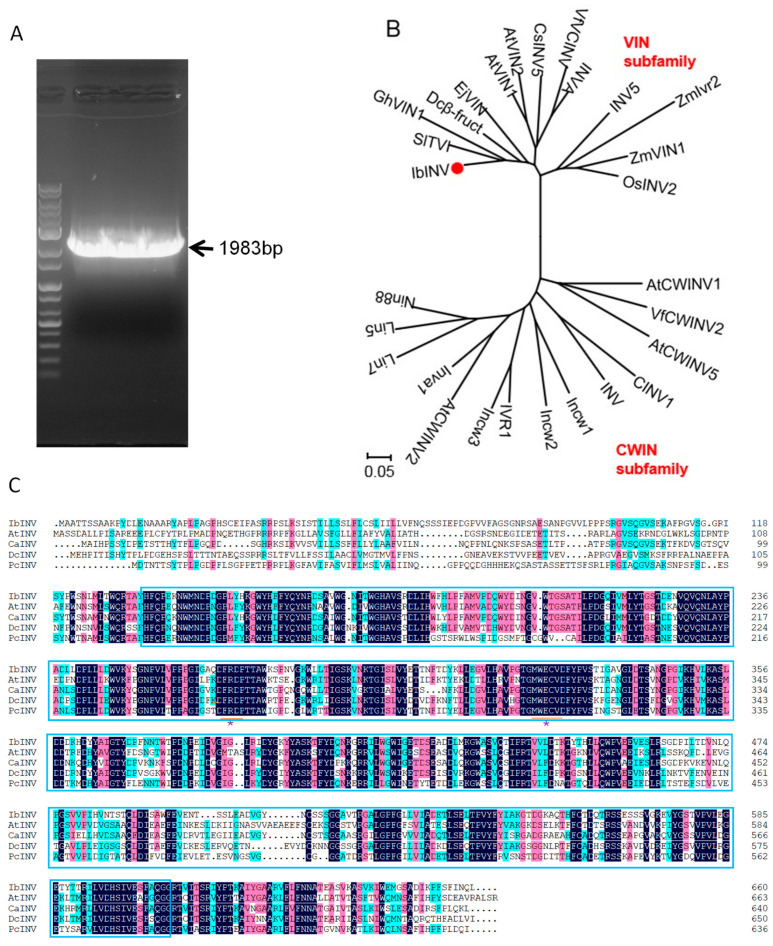
(**A**) The INV-like (IbINV) gene in sweet potato. (**B**) Phylogenetic analysis of sweet potato IbINV gene. The INV sequences used in the analysis are from *Nicotiana tabacum* (accession no: XP_019232131); *Solanum tuberosum* (ADM47340); *Solanum lycopersicum* (NP_001234843); *Cucumis melo* (ABX55832); *Capsicum annuum* (PHT87043); *Gentiana triflora* (BAP47496); *Daucus carota* (CAA53098); *Coffea arabica* (XP_027103944); *Artemisia annua* (PWA88480); *Oryza sativa* (AAD10239); *Cynara cardunculus* var. *scolymus* (XP_024981537); *Sesamum indicum* (XP_011101120); *Helianthus annuus* (OTF99883); *Olea europaea* var. *sylvestris* (XP_022855922); *Lactuca sativa* (XP_023762431); *Dimocarpus longan* (AJW82912); *Camellia sinensis* (XP_028104291); *Actinidia chinensis* (AFO84092); *Nelumbo nucifera* (XP_010259020); *Paeonia lactiflora* (QBA82075); *Gossypium arboreum* (XP_017638224); *Elsholtzia haichowensis* (AFV59227); *Populus trichocarpa* (XP_002303519); *Theobroma cacao* (XP_017972919); *Manihot esculenta* (XP_021615830); *Durio zibethinus* (XP_022737204); *Juglans regia* (XP_018815884). IbINV is marked with red dots; (**C**) Comparison of amino acid sequences of INVs from sweet potato, *Arabidopsis thaliana* (NP_563901.1), *Capsicum annuum* (NP_001311791.1), *Daucus carota* (P_017235973.1) and *Prunus cerasus* (AAL05427.2). Identical and similar amino acid residues are shaded in black and red, respectively. Blue box indicates glycosyl hydrolase family 32 conserved domain. FRDP and WECVD conserved motives are marked by * and orange underline. Background blue, red and black represent different similarity of the sequences.

The IbINV contained a 1980 bp ORF (Figure 1A), which encoded a 660 aa polypeptide with a molecular weight of 72.3 kDa. Phylogenetic analysis of the deduced INV amino acid sequences from 28 species showed that INV could be divided into the VIN and CWIN subfamilies. IbINV has the closest relationship with SlTVI (Figure 1B), both belonging to the VIN subfamily. A comparison of the deduced amino acid sequences of AtINV, CaINV, DcINV, and IbINV revealed that IbINV contained the glycosyl hydrolase family 32 conserved domain and the FRDP and WECVD conserved motives (Figure 1C).

### 2.2. Expression Patterns of IbINV in Various Tissues of Sweet Potato

**Figure 2:**
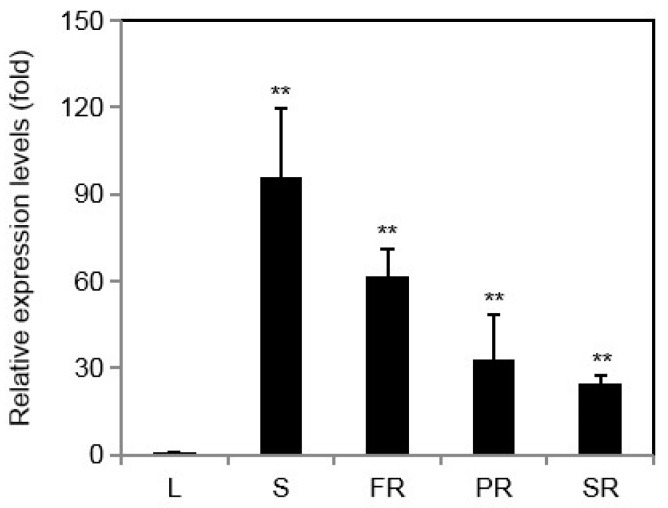
Expression levels of IbINV in different tissues. Fourth leaf from the top (L), stem from fourth–sixth internode (S), fibrous root (FR), pigment root (PR), and storage root (SR) were collected from 2-month-old sweet potato plants. Error bars represent standard deviation (SD) of three independent experiments. Asterisks indicate significant differences from leaf by Duncan’s multiple range test; **: *p* < 0.01.

By qRT-PCR, we examined the relative expression levels of IbINV in different tissues, including the leaf (L), stem (S), fibrous root (FR), pigment root (PR), and storage root (SR). As shown in Figure 2, IbINV transcripts were detected in all analyzed plant tissues. The expression was most abundant in the stem, followed by the fibrous root, pigment root, and storage root, while the expression level of IbINV in leaves was the lowest among the examined tissues.

### 2.3. IbINV Expression Profiles under Various Stresses

**Figure 3:**
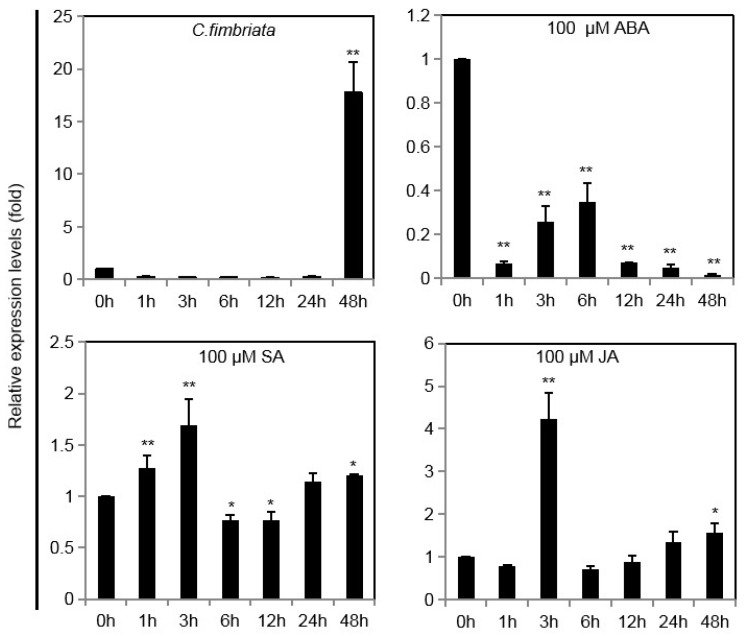
*IbINV* transcripts under biotic and abiotic stresses. Four-week-old sweet potato plants treated with conidiospore suspensions of *C. fimbriata* with concentrations of 1.0 × 10^5^ CFU/mL, 100 μM abscisic acid (ABA), 100 μM salicylic acid (SA), and 100 μM jasmonic acid (JA), respectively, and sampled at 0, 1, 3, 6, 12, 24, and 48 h after treatment. The data shown are the means ± SD of three biological repeats. Asterisks indicate significant differences from 0 h by Duncan’s multiple range test; *: *p* < 0.05; **: *p* < 0.01.

To investigate whether *IbINV* transcript levels could be affected by various stresses, including *C. fimbriata*, ABA, SA, and JA (Figure 3), the relative expression level of *IbINV* in leaves was monitored. In seedlings inoculated with *C. fimbriata*, the relative expression level of *IbINV* was lower than in untreated control plants at 1, 3, 6, 12, and 24 h, but there were no significant differences between them. However, the expression level of *IbINV* was 17.7-fold higher at 48 h after inoculation than that in the untreated control seedlings. The highest expression level of *IbINV* was observed at 3 h after SA and JA treatments, being 1.69-fold and 4.23-fold higher, respectively, than that in the untreated control seedlings. In the ABA-treated seedlings, the transcript levels of *IbINV* were significantly lower than those in the untreated control seedlings. Collectively, these results indicated that *IbINV* could be up-regulated by *C. fimbriata* and SA and JA treatments and down-regulated by ABA.

### 2.4. Generation of Transgenic Sweet Potato Plants with Modified IbINV Expression

To examine whether *IbINV* is involved in the regulation of black rot disease resistance, transgenic sweet potato plants overexpressing *IbINV* (referred to as OEV plants) and transgenic sweet potato plants down-regulating *IbINV* (RiV plants) were generated by *Agrobacterium*-mediated sweet potato callus transformation. Twelve independent OEV plants were confirmed by genome PCR with CaMV35S-F/IbINV-R and CaMV35S-specific primers (Figure S1A), and nine RiV plants were confirmed by genome PCR analysis with CaMV35S-specific primers (Figure S1B). The *IbINV* expression levels of twelve OEV plants and nine RiV plants were identified preliminarily. Two lines of OEV plants (OEV7 and OEV8) showing elevated *IbINV* expression levels and two RiV plants (RiV3 and RiV5) showing reduced *IbINV* expression levels were selected for further characterization (Figure S2A,B).

**Figure 4:**
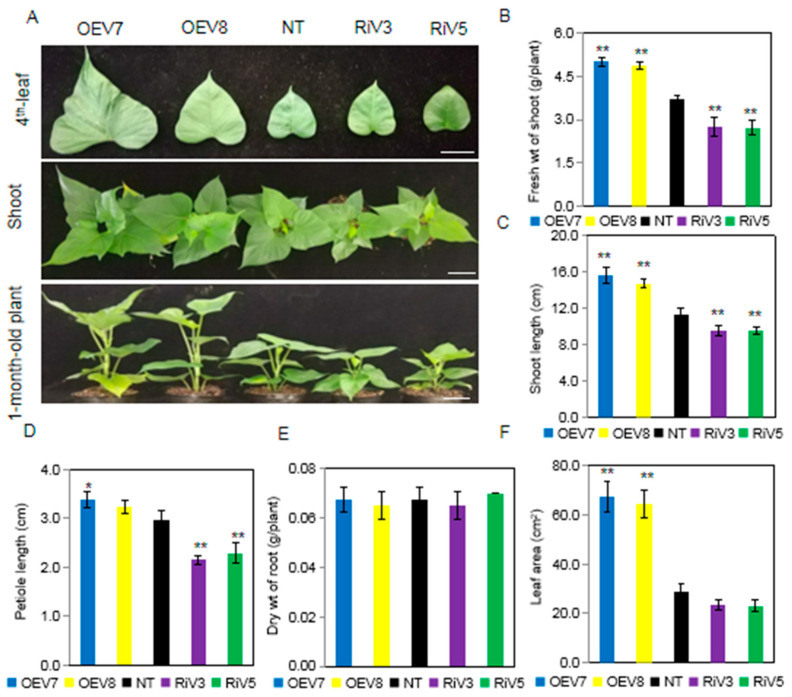
Morphological phenotypes of *IbINV* overexpressing sweet potato plants (OEV) and *IbINV* down-regulating plants (RiV) OEV and RiV plants. (**A**) Plant phenotype of non-transgenic (NT), OEV, and RiV plants after 1 month growth in pots. The bar means 3 cm. (**B**) Fresh weight of shoot. (**C**) Shoot length. (**D**) Petiole length. (**E**) Dry weight of root. (**F**) Leaf (4th leaves of 1-month-old sweet potato plants) area. The data shown are the means ± SD of three biological repeats. Asterisks indicate significant differences from NT plants by Duncan’s multiple range test; *: *p* < 0.05; **: *p* < 0.01.

One-month-old OEV plants produced increased shoot biomass (Figure 4A). The average fresh weights of shoots were 5.00 and 4.86 g/plant in OEV7 and OEV8 plants, respectively. However, the average fresh weight of shoots was 2.75 and 2.72 g/plant in RiV3 and RiV5 plants, respectively. The shoot biomass in both of the OEV plants and RiV plants was completely different and significantly higher or lower compared to NT plants (Figure 4B). The shoot length, petiole length, and leaf area showed similar results, reflecting the phenotypes in OEV, NT, and RiV plants (Figure 4C,D,F). However, there was no significant difference in the dry weight of roots in OEV plants and RiV plants compared to NT plants (Figure 4E).

### 2.5. Resistance to Black Rot Disease of Transgenic Sweet Potato Plants

**Figure 5:**
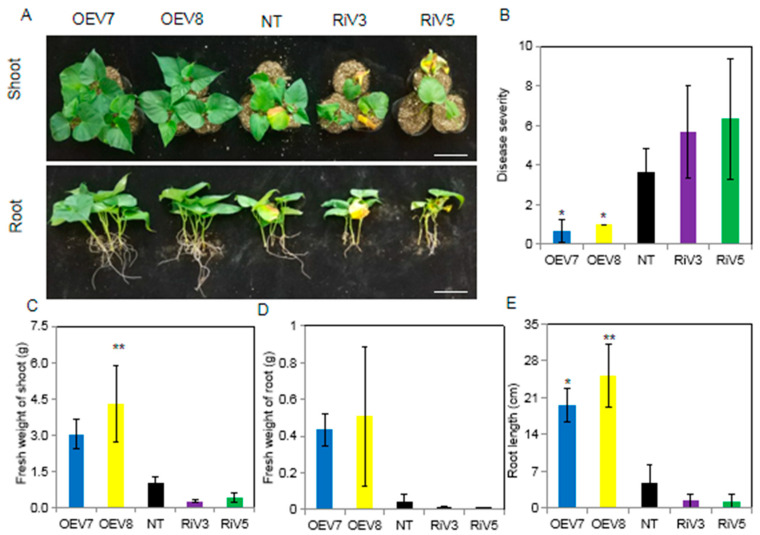
Resistance identification of the transgenic sweet potato plants (OEV and RiV plants) and NT against black rot disease of sweet potato. (**A**) Phenotypes of 1-month-old NT, OEV, and RiV plants after treatment with *C. fimbriata* for 15 days. The bar means 8 cm. (**B**) Disease severity. (**C**) Fresh weight of shoot. (**D**) Fresh weight of root. (**E**) Root length of NT, OEV, and RiV plants after *C. fimbriata* inoculation for 15 days. Data represent the mean ± SD of three biological replicates. Asterisks indicate significant differences from NT plants by Duncan’s multiple range test; *: *p* < 0.05; **: *p* < 0.01.

After inoculating *C. fimbriata* for 15 days, we observed that the resistance to black rot disease was significantly increased in OEV plants compared to NT plants, while the resistance to black rot disease was decreased in RiV plants compared to NT plants (Figure 5A). The disease severity was significantly reduced in OEV plants at 0.67 and 1.00, which was 5.48-fold and 3.67-fold lower than in NT plants, respectively (Figure 5B). The fresh weights of shoot and root length were significantly higher in OEV plants than in NT and RiV plants (Figure 5C,E). It was clearly observed from the phenotype that the roots of OEV plants branched and grew normally, while the roots of RiV and NT plants were rarely branched and showed rot symptoms. The root fresh weight was measured, and there was no significant difference between the transgenic lines and the NT plants (Figure 5D). The root length was significantly longer in OEV plants compared to NT and RiV plants (Figure 5E).

### 2.6. Invertase Activity in Transgenic Sweet Potato Plants

**Figure 6:**
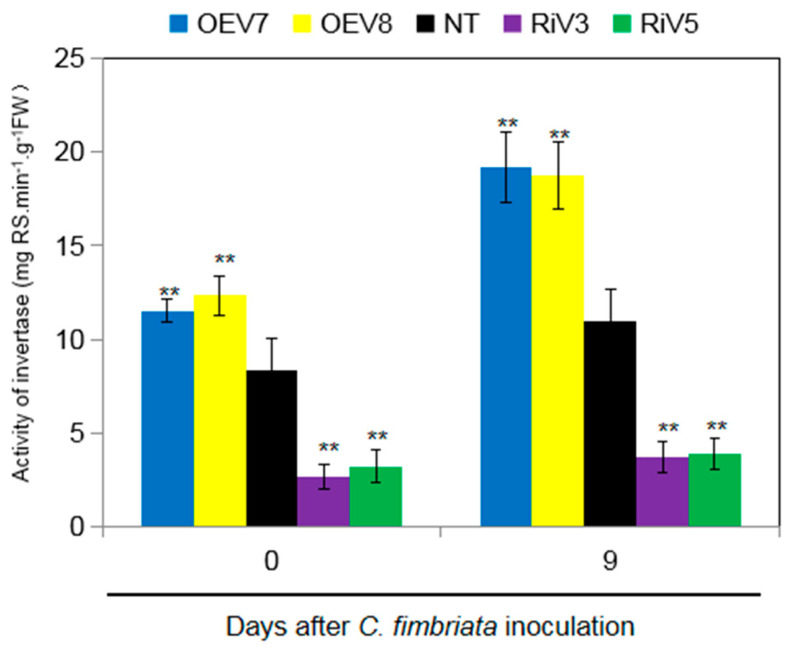
Activity of invertase in leaves of transgenic sweet potato plants (OEV and RiV) and NT plants before inoculation and after treatment with *C. fimbriata* for 9 days. Data represent the mean ± SD of three biological replicates. Asterisks indicate significant differences from NT plants by Duncan’s multiple range test; **: *p* < 0.01.

The invertase activities were determined (Figure 6). Before inoculation, the invertase activity of OEV plants was significantly higher than that of NT plants, while it was significantly lower in RiV plants than that of NT plants. Nine days after *C. fimbriata* inoculation, the invertase activities increased in general, with higher activity observed in the OEV plants than in the NT and RiV plants.

### 2.7. Sugar Content in Transgenic Sweet Potato Plants

**Figure 7:**
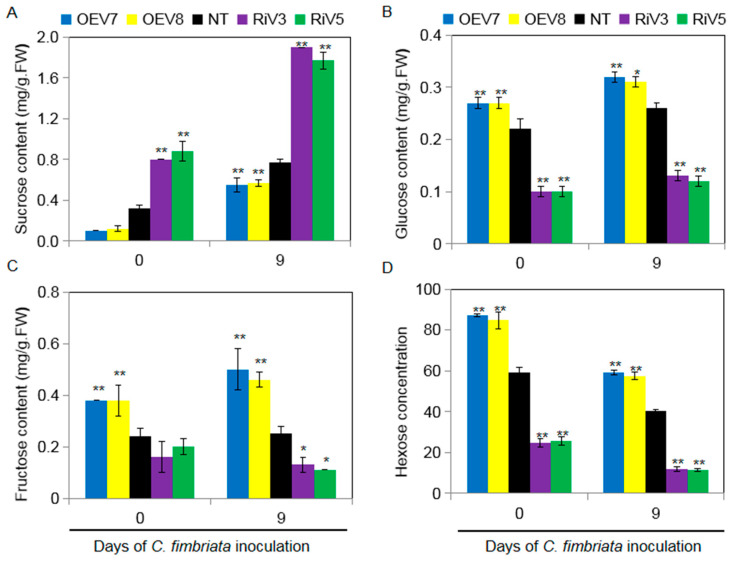
Sugar (sucrose, glucose, and fructose) contents in leaves of transgenic sweet potato plants (OEV and RiV) and NT plants before and after inoculation with *C. fimbriata* for 9 days. (**A**) Sucrose content. (**B**) Glucose content. (**C**) Fructose content. (**D**) Hexose concentration [(glucose + fructose)/(sucrose + glucose + fructose)] × 100. The data shown are the means ± SD of three biological repeats. Asterisks indicate significant differences from NT plants by Duncan’s multiple range test; *: *p* < 0.05; **: *p* < 0.01.

To investigate the involvement of sugar metabolism in black rot disease resistance, the sugar contents in the leaves of transgenic sweet potato after *C. fimbriata* inoculation were determined. Before *C. fimbriata* inoculation, the sucrose contents were lower in OEV plants, while glucose and fructose contents were higher compared with those in NT plants. In contrast, the RiV plants showed the opposite effects. Sucrose, glucose, and fructose contents increased in OVE and NT plants at 9 days after *C. fimbriata* post-inoculation, especially for sucrose contents (Figure 7A–C). The total hexose content in the leaves of RiV plants was lower than in NT and OEV plants before and after the *C. fimbriata* inoculation (Figure 7D).

## 3. Discussion

As an important energy and signal molecule in plants, sucrose cannot be directly utilized by cells, and it can only be absorbed and utilized after being converted into glucose and fructose [36]. Invertase is a hydrolase that catalyzes the hydrolysis of sucrose to glucose and fructose irreversibly, thus ensuring the supply of carbon and energy in plants. In plants, the function of invertase is very complex, which not only provides carbon for sink and source but also participates in the distribution of sucrose in the early stage of plant development [37]. This enzyme plays a very important role, not only in plant growth and development, but also in resistance to various stresses [30]. However, it is still largely unknown how INV is involved in the regulation of plant growth and the response to biotic stress. In this study, we identified for the first time an *INV* homolog (*IbINV*) in sweet potato and characterized its roles in sucrose metabolism, plant growth, and black rot disease resistance.

At present, 9, 16, and 8 potential neutral/alkaline invertase sequences have been isolated from *Arabidopsis*, poplar, and rice, respectively [38]. Previous studies have shown that invertases belong to the glycosyl hydrolase 32 (GH32) gene family [39]. Cell wall invertase and vacuolar invertase have high enzymology similarity and high sequence similarity. Its amino acid sequence contains three conserved motifs (motifs), such as NDPNG/A, FRDP, and WEC (P/V) D, which are important parts of the active site of sucrose invertase [40]. Although cell wall invertase and vacuolar invertase are highly similar in enzymatic properties and sequence, they can be distinguished by three main characteristics: cluster analysis with a 100% highly significant bootstrap value; sequence alignment showed that vacuolar invertase usually has N-terminal extension, which may be related to targeted vacuoles; and the conservative motif WEC (P/V) D determines the different optimal pH values of the two invertases [41]. In the cell wall invertase, the fourth position of WEC (P/V) D is proline residue (P), while in the vacuolar invertase, the fourth position of the motif is valine residue (V) [42]. In the current study, computer analysis revealed that IbINV contains a glycosyl hydrolases family 32 conserved domain, which showed that it belongs to a member of glycosidase family GH32. Furthermore, IbINV also contains FRDP and WECVD motifs, while the NDPNG motif mutated to NDPDG (Figure 1). In addition, sweet potato plants overexpressing *IbINV* increase sucrose hydrolysis into hexoses, whereas silencing the expression of the *IbINV* led to a decrease in sucrose hydrolysis into hexoses (Figure 7). These results clearly prove that IbINV is functionally conserved with other plant INVs.

Sugars are not only immediate substrates for intermediary metabolism, but they are also effective signaling molecules in plants. Generally, sugar availability is linked to plant growth and development, but how exactly sugars control growth and development is still unknown. Some evidence suggests that the hydrolysis of sucrose by invertase is related to growth and cell expansion by providing “sink strength” for sucrose transport from source leaves into sink organs (phloem unloading) [43]. For example, in carrots, suppressing the expression of *DcCWIN1* prevented the tap roots formation, and the levels of soluble sugars and starch were significantly reduced in the small primary-type roots [44]. Suppressing the expression of *OsCWIN2* in rice reduced grain yield, whereas enhancing the expression of *OsCWIN2* achieved a contrary result [45]. Silencing the expression of *SlCWIN1* in tomato inhibited seed and fruit development, while elevating its activity showed the opposite effect [46]. In the overexpression of *GmCInv Arabidopsis*, the rosette leaf diameter was significantly larger than that of the wild type, indicating more vigorous growth of the above-ground parts [47]. Antisense suppression of an acid invertase gene reduced plant growth and fruit development in muskmelon [48]. These findings illustrate that invertase plays a crucial role in sucrose hydrolysis and regulates the growth and development of plants. In this study, up-regulating the expression of *IbINV* in sweet potato (OEV plants) elevated the invertase activity and consequently led to higher hexoses (glucose and fructose) but lower sucrose content (Figure 6 and Figure 7). The OEV plants displayed elongated shoot length and petiole length, increased fresh weight of shoots, and larger leaves, but no significant difference was observed in root dry weight. However, after the initiation of storage root, the aboveground part of OEV plants became similar to that of NT and RiV plants (Figure 4 and Figure 5). These could be caused by the transportation of organic substances from leaves to sink organs.

After being infected by pathogens, plants will trigger a series of defense reactions to resist their infection, including reactive oxygen species production, hypersensitive response reactions, pathogenesis-related protein synthesis, plant protection synthesis, reconstruction of the cytoskeleton, and the thickening of cell walls [49]. Most of the above reactions occur within a few hours after the pathogen infects the plant. These reactions require a lot of energy in the plant. Sucrose is not directly involved during the process of plant disease resistance, but it is decomposed into hexose by invertase and regulates the sugar signal process through the change in hexose concentration, thereby triggering the plant’s defense response [50]. Thus, sucrose is not only seen as a source of energy for plants but also as an important signaling factor in the process of plant disease resistance. Many experiments have shown that the increase in sugar content plays a crucial role in the interaction between plants and pathogens [51]. The overexpression of yeast invertase in tobacco increases the accumulation of hexose and also increases resistance to viruses. Reducing the activity of invertase in tobacco leaves by RNA interference technology led to the reduced accumulation of callose between cells, the output of sugar, and the extracellular carbohydrates related to defense, which would cause the activity of phenylpropionate deaminase and hexadecanoate glucose dehydrogenase to be reduced, the formation of peroxides to be damaged, and the hypersensitivity reaction to be weakened; thus, the leaves could not effectively prevent the spread of the virus [52]. In this study, OEV plants showed high resistance to black rot disease compared to NT plants 15 days after *C. fimbriata* infection, while RiV plants showed a completely contrary result, which obviously illustrated that IbINV positively regulated resistance to black rot disease caused by *C. fimbriata* in sweet potato (Figure 5). The increased activity of invertase in OEV plants regulated the sugar signal process through the change in the hexose concentration and thereby triggered the plant’s defense response, which may be a possible mechanism for IbINV-mediated black rot disease resistance (Figure 6 and Figure 7).

## 4. Materials and Methods

### 4.1. Plant and Fungal Strains

*Ipomoea batatas* (L.) Lam. cv. Xushu 29 plants were used in this study. The methods previously described by Kang et al. for plant cultivation, callus inducing, and subculturing were used [53]. To analyze tissue-specific expression of *IbINV*, leaves (the 4th fully expanded leaves from the tops of plants), stems (4th–6th internode), fibrous roots, pigment roots, and storage roots were collected from 10-week-old sweet potato plants and immediately frozen with liquid nitrogen.

The *C. fimbriata* strain was previously isolated from diseased tubers, which were collected from the field, identified following Koch’s postulates, and stored in the Jiangsu Xuzhou Sweet Potato Research Center. The pathogen was grown in a PDA medium (200 g/L potato, 20 g/L dextrose, and 16 g/L agar powder) for approximately 5 days. Then, the mycelium was washed with sterile water, and the concentration of spores was adjusted to 1 × 10^5^ CFU/mL.

### 4.2. IbINV Cloning and Phylogenic Analysis

Total RNA was extracted from the fourth fully expanded leaves of Xushu 29 using TRIzol reagent (Invitrogen, Carlsbad, CA, USA), according to the manufacturer’s instructions. Two micrograms of total RNA were reverse transcribed using the Prime Script TM II 1st Strand cDNA Synthesis Kit (TakaRa, Dalian, China) to synthetize the 1st strand cDNA. The sweet potato *INV* (*IbINV*) gene was identified by sequence comparison with the potato INV. The coding sequence of *IbINV* was amplified using the KOD Plus-Neo Kit (Toyobo, Osaka, Japan). The specific primer pairs are shown in Table 1. The PCR products were purified by agarose gel electrophoresis, the sequence was confirmed, and then they were cloned into the T-blunt vector (BioFACT, Daejeon, Republic of Korea).

Nucleotide sequences of *INV* family members were retrieved from the US National Center for Biotechnology Information (NCBI) and converted to amino acid sequences using BioEdit. Multiple sequence alignments were carried out using BioEdit. Phylogenetic trees were constructed using MEGA6. The neighborhood-joining method was used to create the phylogenetic trees with 1000 bootstrap replicates. The values in phylogenic tree reflect the evolutionary distances, which were computed using the Poisson correction method [54].

### 4.3. Recombinant Plasmid Construction

According to the Gateway cloning technology, the attB site-containing stop-codon-less ORF sequence of *IbINV* and attB site-containing fragment from 1634–1932 bp of *IbINV* cDNA were amplified and then generated as entry vectors based on pDONR207 using BP Clonase (Invitrogen, Carlsbad, CA, USA). IbINV-S was identified using the BLOCK-iTTM RNAi Designer (Invitrogen, Carlsbad, CA, USA). The specific primer pairs are shown in Table 1.

For overexpressing *IbINV* in sweet potato, the ORF sequence of *IbINV* was cloned into the destination vector PGWB5 using LR Clonase (Invitrogen, Carlsbad, CA, USA) to create the overexpressing recombinant plasmid. To construct the IbINV RNAi vector, IbINV-S was cloned into the destination vector pH7GWIWG2(I) using LR Clonase (Invitrogen, Carlsbad, CA, USA) to create the corresponding RNAi recombinant plasmid.

### 4.4. Sweet Potato Transformation for Generation of Transgenic Plants

*IbINV*-overexpressing and *IbINV*-RNA interference-expressing sweet potato plants were separately generated by the *Agrobacterium*-mediated transformation of Xushu 29 embryogenic callus, as described previously [54]. To confirm that the regenerated lines were transgenic lines, genomic DNA and total RNA were extracted, and identification of transgenic plants was performed by PCR and qRT-PCR. Non-transgenic sweet potato plants served as the negative controls. The specific primers are shown in Table 1.

### 4.5. C. fimbriata Inoculation and Stress Treatment

For conidiospore suspension of *C. fimbriata* treatment, four-week-old sweet potato plants were inoculated with *C. fimbriata* (1 × 10^5^ CFU/mL). The leaves were collected at 0, 1, 3, 6, 12, 24, and 48 h after treatment and immediately frozen with liquid nitrogen.

For abiotic stress treatments, the plants were grown in chamber at 25 °C with a 16 h light/8 h dark photoperiod for four weeks and then treated with 100 μM of abscisic acid (ABA), 100 μM of salicylic acid (SA), and 100 μM of jasmonic acid (JA). The leaves were collected at 0, 1, 3, 6, 12, 24, and 48 h after treatment and immediately frozen with liquid nitrogen.

### 4.6. Gene Expression Analysis

To analyze the mRNA expression profiles under different stresses and the tissue-specific expression pattern of *IbINV*, total RNA was extracted with Trizol reagent, as described in Section 4.2. For the 1st strand cDNA synthesis, 2 μg of total RNA was reverse transcribed using TopScript™ RT DryMIX (dT18) (Enzynomic, Daejeon, Republic of Korea), following the manufacturer’s instructions. The reaction mixture was diluted to 100 μL with sterilized water. The quantitative RT-PCR analysis was performed with a Bio-Rad CFX96 thermal cycler using Ever-Green 20 fluorescent dye (BioFACT, Daejeon, Republic of Korea). The *IbACTIN* gene was used as the reference gene. The specific primer sequences for qRT-PCR are listed in Table 1. The gene expression was quantified using the 2^−ΔΔCT^ method. The experiments were conducted with three biological replicates, each with three plants.

### 4.7. Black Rot Disease Resistance Assay

Four-week-old seedlings were cut down and planted in a pot filled with sterile soil, and 50 mL of *C. fimbriata* (1 × 10^5^ CFU/mL) was inoculated through the basal part of the stem into each pot. Sterile water was sprayed once a day to keep moist for black rot disease occurrence. The incidence of black rot disease was determined 15 days after inoculation. Disease severity of black rot disease was assessed using the following scales: 0, no symptoms; 1, normal rooting, scattered black spots on the stem base of the seedlings, accompanied with yellow or wilting leaves, normal branching; 3, reduced rooting, black spots on the stem base of the seedlings, accompanied with yellow or wilting leaves, slowly growing; 5, no rooting, black spots on the stem base of the seedlings, accompanied with yellow or wilting leaves, no branching; 7, root necrosis, with wilting and yellow leaves of the whole plant, no branching; and 9, the whole plant is necrosis and rotten. The experiments were conducted with three biological replicates, each with three plants. Disease severity was calculated as the following formula: Disease severity = [∑ (number of diseased plants in this index × disease index)/(total number of plants investigated × the highest disease index)] × 100%.

### 4.8. Sugar Measurement

Fresh leaves (0.1 g) were sampled from *C. fimbriata*-inoculated sweet potato plants. Samples were collected before infection and 9 days after inoculation. Briefly, the samples were ground to a fine powder, then weighed and incubated with 80% ethanol at 80 °C for 2 h. The supernatant was collected for measurements of sugar content. Sucrose, glucose, and fructose were measured using corresponding Assay Kits (Solarbio, Beijing, China), according to the manufacturer’s instructions. The experiments were conducted with three biological replicates for each treatment.

### 4.9. Analysis of Invertase Activity

Fresh leaves (0.1 g) were sampled from *C. fimbriata*-inoculated sweet potato plants. Samples were collected before infection and 9 days after inoculation. Activities of invertase enzyme were measured using Acid Invertase Assay Kits (Solarbio, Beijing, China), following the manufacturer’s instructions. The experiments were conducted with three biological replicates for each treatment.

### 4.10. Statistical Analysis

Data were statistically analyzed using Statistical Package for the Social Sciences (SPSS 19.0, SPSS Inc., Chicago, IL, USA). Means were separated using Duncan’s multiple range test at *p* = 0.05 and *p* = 0.01.

## 5. Conclusions

In this study, we successfully developed transgenic sweet potato plants overexpressing *IbINV*, which displayed vigorous plant growth and enhanced black rot disease resistance, whereas transgenic sweet potato plants down-regulating the expression of *IbINV* showed reduced growth and black rot disease sensitivity. Although further studies are needed to understand the exact relationship between sucrose metabolism and black rot disease infection, this study provides a new gene for developing black rot-disease-resistant sweet potato plants.

## Figures and Tables

**Table 1:** List of gene-specific primers used in this study.

Primer Name	Sequence (5′-…-3′)	Application
IbINV_F	ATGGCCGCCACCACTTCTTCCG	*IbINV* isolation
IbINV_R	TTACAATTGATTGATGAAAGAG	*IbINV* isolation
IbINV_attb_F	AAAAAGCAGGCTGCATGGCCGCCACCACTTCT	attB-*IbINV*
IbINV_attb_R	AGAAAGCTGGGTCCAATTGATTGATGAAAGAG	attB-*IbINV*
IbINV-S_attb_F	AAAAAGCAGGCTGCATATCGCAAAGGGCACC	attB-IbINV-S
IbINV-S_attb_R	AGAAAGCTGGGTCCCAGATCTTCACCGACG	attB-IbINV-S
RTIbINV_F	GGGGCCGTTCGGACTTCT	RT-PCR for *IbINV*
RTIbINV_R	ACCGTGCTTCCATAAACCTCTT	RT-PCR for *IbINV*
*Actin*-F	AGCAGCATGAAGATTAAGGTTGTAGCAC	Reference gene
*Actin*-R	TGGAAAATTAGAAGCACTTCCTGTGAAC	Reference gene

## Data Availability

Data are contained within the article and Supplementary Materials.

## Supplementary Materials

The following supporting information can be downloaded at: https://www.mdpi.com/article/10.3390/ijms242216454/s1.

## Abbreviations

INV: invertase; ABA, abscisic acid; SA, salicylic acid; JA, jasmonic acid; OEV plants, transgenic sweet potato plants overexpressing *IbINV*; RiV plants, transgenic sweet potato plants down-regulating *IbINV*; qRT-PCR, real-time quantitative PCR; CWIN, cell wall invertases; VIN, vacuolar invertases; L, leaf; S, stem; FR, fibrous root; PR, pigment root; SR, storage root.
